# Existence of SARS-CoV-2 Entry Molecules in the Oral Cavity

**DOI:** 10.3390/ijms21176000

**Published:** 2020-08-20

**Authors:** Wakako Sakaguchi, Nobuhisa Kubota, Tomoko Shimizu, Juri Saruta, Shinya Fuchida, Akira Kawata, Yuko Yamamoto, Masahiro Sugimoto, Mayumi Yakeishi, Keiichi Tsukinoki

**Affiliations:** 1 Division of Environmental Pathology, Department of Oral Science, Kanagawa Dental University, 82 Inaoka, Yokosuka, Kanagawa 238-0003, Japan; sakaguchi@kdu.ac.jp (W.S.); n.kubota@kdu.ac.jp (N.K.); mhori77@ykh.gr.jp (M.Y.); tsukinoki@kdu.ac.jp (K.T.); 2 Department of Highly Advanced Oral Medicine, Kanagawa Dental University, 3-31-6 Tsuruya-cho, Yokohama, Kanagawa 221-0835, Japan; shimizu@kdu.ac.jp; 3 Division of Dental Sociology, Department of Disaster Medicine and Dental Sociology, Kanagawa Dental University, 82 Inaoka, Yokosuka, Kanagawa 238-0003, Japan; fuchida@kdu.ac.jp; 4 Division of Histology, Embryology and Neuroanatomy, Department of Oral Science, Kanagawa Dental University, 82 Inaoka, Yokosuka, Kanagawa 238-0003, Japan; kawata@kdu.ac.jp; 5 Division of Dental Hygiene, Kanagawa Dental University Junior College, 82 Inaoka, Yokosuka, Kanagawa 238-0003, Japan; yamamoto.yuko@kdu.ac.jp; 6 Research and Development Center for Minimally Invasive Therapies, Medical Research Institute, Tokyo Medical University, 6-1-1 Shinjuku, Tokyo 160-8402, Japan; mshrsgmt@tokyo-med.ac.jp

**Keywords:** SARS-CoV-2, oral cavity, saliva, tongue coating, taste cell, ACE2, TMPRSS2, furin

## Abstract

The severe acute respiratory syndrome coronavirus 2 (SARS-CoV-2) receptor, angiotensin-converting enzyme 2 (ACE2), transmembrane protease serine 2 (TMPRSS2), and furin, which promote entry of the virus into the host cell, have been identified as determinants of SARS-CoV-2 infection. Dorsal tongue and gingiva, saliva, and tongue coating samples were examined to determine the presence of these molecules in the oral cavity. Immunohistochemical analyses showed that ACE2 was expressed in the stratified squamous epithelium of the dorsal tongue and gingiva. TMPRSS2 was strongly expressed in stratified squamous epithelium in the keratinized surface layer and detected in the saliva and tongue coating samples via Western blot. Furin was localized mainly in the lower layer of stratified squamous epithelium and detected in the saliva but not tongue coating. ACE2, TMPRSS2, and furin mRNA expression was observed in taste bud-derived cultured cells, which was similar to the immunofluorescence observations. These data showed that essential molecules for SARS-CoV-2 infection were abundant in the oral cavity. However, the database analysis showed that saliva also contains many protease inhibitors. Therefore, although the oral cavity may be the entry route for SARS-CoV-2, other factors including protease inhibitors in the saliva that inhibit viral entry should be considered.

## 1. Introduction

Coronavirus disease 2019 (COVID-19), caused by the severe acute respiratory syndrome coronavirus 2 (SARS-CoV-2), is an emerging infectious disease that has spread worldwide [1]. In general, the oral cavity is an important entry point for pathogens. Furthermore, since SARS-CoV-2 is found in saliva, attempts have been made to use saliva samples in PCR tests for COVID-19, and there has been a great deal of interest in the oral cavity and saliva in associated research [2]. However, few basic studies have been conducted on the association between the oral cavity and COVID-19.

Using mainly database mining, Xu et al. reported the expression of angiotensin-converting enzyme 2 (ACE2) in the oral mucosa, especially the dorsal tongue [3]. ACE2 is a host receptor to which SARS-CoV-2 binds [3]. Hamming et al. reported that ACE2 is localized in the basal layer of non-keratinizing stratified squamous epithelium, but these findings are not described in detail [4]. The expression of ACE2 in the taste buds remains unclear, although taste impairment has received particular attention as a symptom of COVID-19 [5].

It is presumed that SARS-CoV-2 has multiple pathways of entry into human cells [6]. However, the receptor–protease-mediated pathway of entry is important because it enhances viral infectivity [7]. Transmembrane protease serine 2 (TMPRSS2) has been reported to be an important protease for SARS-CoV-2 invasion [8]. TMPRSS2 is a serine protease strongly expressed in the prostate gland and is a promising camostat mesylate target for COVID-19 treatment [9]. SARS-CoV-2 uses spike protein (SP) to bind the cell membrane, a process activated by specific cellular enzymes such as furin [10]. Coutard et al. revealed that the SP of SARS-CoV-2 possesses a furin-like cleavage site, which is absent in other CoVs of the same clade [11]. The localization of host receptors and these proteases is therefore a critical determinant of viral infection. Interestingly, ACE2 and TMPRSS2 have been shown to be expressed genes in various organs throughout the body, including the lung, heart, bladder, pancreas, kidney, skin, and small intestine [12,13]. However, there have been few immunohistochemical and molecular biological studies focused on these factors in the oral cavity [14].

In addition to the tongue, studies on the oral cavity should also focus on other elements of the oral environment such as the saliva and tongue coating. The gingival sulcular epithelium is stratified squamous epithelium with no keratinization, very thin, affected by inflammation, and a site where bacteria can easily enter. Therefore, in this study, we clarified the localization of ACE2 in the dorsal tongue tissue and previously unreported periodontal tissues. Furthermore, the presence of several proteases was clarified using tissues, tongue coating, and saliva as search targets. This was mainly focused on TMPRSS2, which is important for SARS-CoV-2 invasion. In this study, we aimed to elucidate the presence of determinants of infection by SARS-CoV-2 in the oral cavity.

## 2. Results

### 2.1. Immunohistochemistry

#### 2.1.1. Tongue Squamous Epithelium

**Figure 1:**
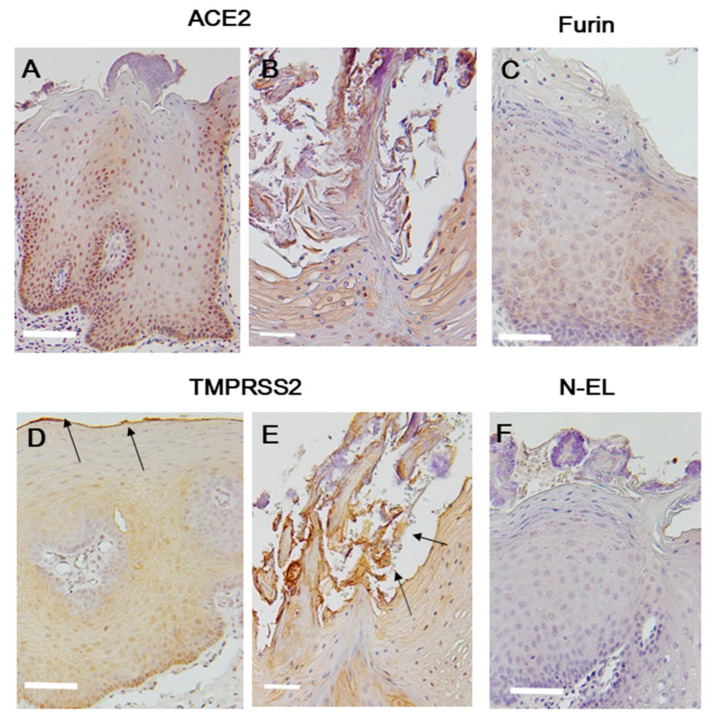
ACE2: Tongue epithelium staining largely in the spinous and basal cell layers (**A**). The upper layer of the tongue epithelium shows bacteria and strong positive staining within cells (**B**). Furin: Tongue epithelium showing consistent staining in the spinous and basal cell layers (**C**). TMPRSS2: Tongue epithelium showing positive staining (**D**). The upper layer of the tongue epithelium shows bacteria and strong positive staining within cells (**E**). N-EL: No immunostaining signals (**F**). The arrows indicate a strong immunochemical positive signal. Scale bar = 50 µm. ACE2, angiotensin-converting enzyme 2; TMPRSS2, transmembrane protease serine 2; N-EL, neutrophil-elastase.

ACE2 expression was mainly observed in the nuclei and cytoplasm of the spinous and basal cell layers (Figure 1A). In the horny layer, ACE2 expression was inconsistent and sporadically observed as a relatively strong signaling region. The localization of ACE2 differed through layers. Although the spinous–basal cell layer showed ACE2 signals in the nucleus and cytoplasm, in the horny layer, the signal was not in the nucleus, but the cytoplasm and cell membrane (Figure 1B). Degenerate cells that disengaged from the epithelium with a microbacterial colony gave an immunohistochemically strong ACE2-positive signal.

Furin was mainly localized in the cytoplasm of the spinous–basal cell layer (Figure 1C). Immunohistochemical signals for furin were observed in a punctate pattern and were more intense in basal cells than in spinous cells. No furin-positive signals were noted in the epithelial surface, horny layer, or degenerate cells.

The epithelial surface, horny layer, and spinous–basal cell layer were positive for TMPRSS2 (Figure 1D arrow). A TMPRSS2-specific signal was observed in the epithelial surface (Figure 1D). The signal in the horny layer was weak, although there was a clear expression in the cell membrane. However, degenerate cells had a strong signal for TMPRSS2 (Figure 1E arrow). Signals in spinous cells were predominantly localized in the cell membrane. Basal cells were largely negative for TMPRSS2, and any positive signals were localized in the cytoplasm.

Neutrophil-elastase (N-EL) signals were completely negative in the spinous and basal cells (Figure 1F). The epithelial surface and horny layer had equivocal expression patterns. Proportion of positive cell is shown in Table 1.

Lymph node B cells were used as a negative control (Figure S1) and no staining was observed with any of the primary antibodies (Figure S2).

#### 2.1.2. Taste Buds

**Figure 2:**
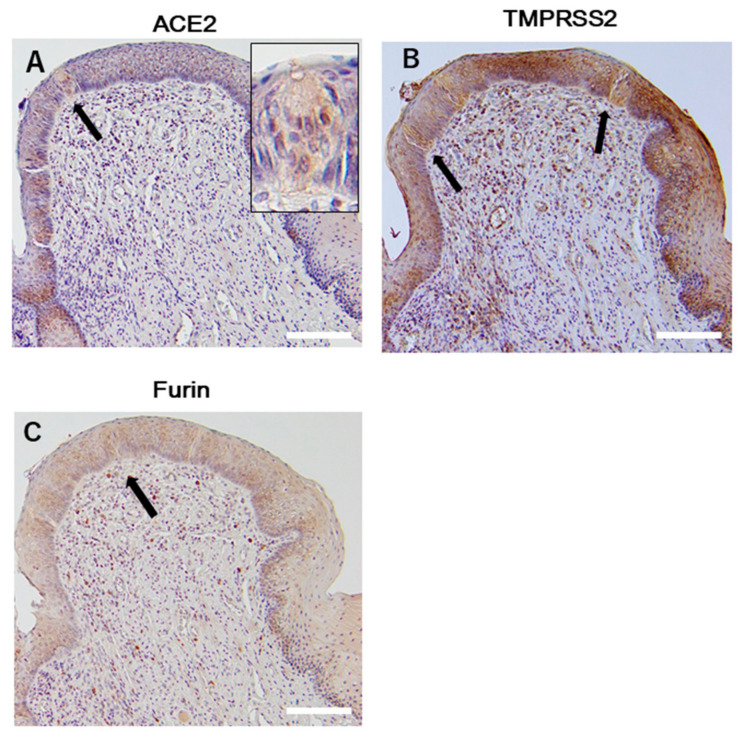
The fungiform papilla of the tongue epithelium. Immunohistochemical staining of ACE2 (**A**), TMPRSS2 (**B**), and furin (**C**) in the tongue epithelium, including the taste buds. High magnification image showing ACE2-positive taste cells in the inset. The arrows indicate the taste bud cells. Scale bar = 100 µm. ACE2, angiotensin-converting enzyme 2; TMPRSS2, transmembrane protease serine 2.

ACE2 protein was consistently expressed in the tongue epithelial layer of the taste buds (Figure 2A arrow). TMPRSS2 expression was strongly positive in taste bud cells (Figure 2B arrow), and furin-positive signals were detected in the lower layers of the taste buds (Figure 2C arrow). Taste buds tended to have stronger signals than the area surrounding epithelial cells. No N-EL staining was detected (data not shown).

#### 2.1.3. Gingival Squamous Epithelium

**Figure 3:**
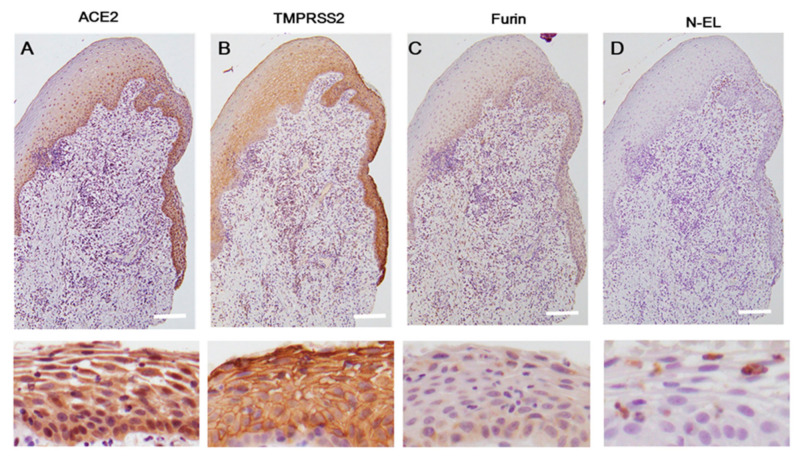
The gingival tissues, including the sulcus epithelium. Immunohistochemical expression of ACE2 (**A**), TMPRSS2 (**B**), and furin (**C**). No N-EL staining was detected in the gingival squamous epithelium (**D**). High magnification images showing ACE2, TMPRSS2, and furin-positive cells in the gingival squamous epithelium and an N-EL-positive signal in neutrophils. Scale bar = 100 µm. ACE2, angiotensin-converting enzyme 2; TMPRSS2, transmembrane protease serine 2; N-EL, neutrophil-elastase.

ACE2 expression was observed in the nucleus and cytoplasm of the spinous–basal cell layer, but not the epithelial surface and horny layer. The buccal surfaces of gingival sulcular epithelium tended to have stronger ACE2 expression than the buccal surfaces of gingival epithelium (Figure 3A).

TMPRSS2 was localized in the epithelial surface, horny layer, and spinous–basal cell layer. A clear TMPRSS2 signal was observed in the epithelial surface in a line-like pattern (Figure 3B).

TMPRSS2 showed largely weak expression in the cell membrane of cells in the horny layer. In spinous cells, TMPRSS2 was localized in the cell membrane and cytoplasm. Although TMPRSS2 signals were localized in the cytoplasm of basal cells, TMPRSS2-negative cells were focally observed. Stronger TMPRSS2-positive signals were observed in the buccal surfaces of sulcular epithelium than in the buccal surfaces of gingival epithelium. Furin-positive signals were observed in a dot-like pattern and were more intense in basal cells than in spinous cells. No furin expression was observed in the cell membrane (Figure 3C). Furin had stronger expression in the buccal surfaces of sulcular epithelium than in the buccal surfaces of gingival epithelium. No furin-positive signals were noted in the epithelial surface and horny layer. No staining for N-EL was observed in the section analyzed (Figure 3D). However, neutrophils infiltrating the intraepithelium had positive signals for N-EL antigen. Positive cell rate is shown in Table 1.

#### 2.1.4. Submandibular Glands

**Figure 4:**
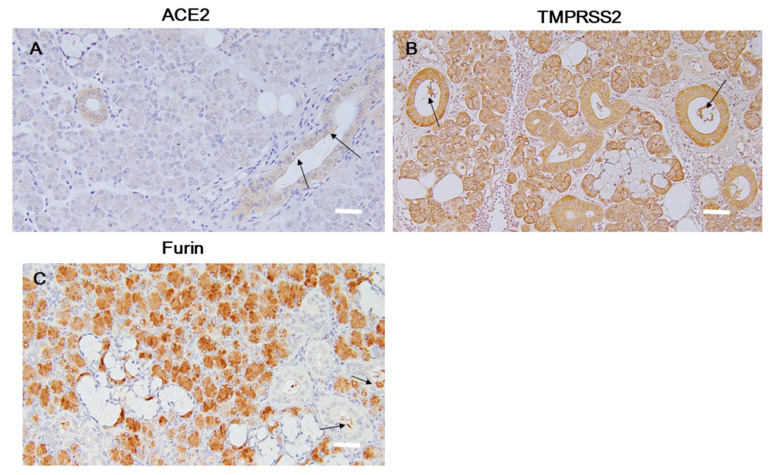
The submandibular gland. Immunohistochemical expression of ACE2 (**A**), TMPRSS2 (**B**), and furin (**C**). The arrows indicate a strong immunochemical positive signal. Scale bar = 100 µm. ACE2, angiotensin-converting enzyme 2; TMPRSS2, transmembrane protease serine 2.

Serous cells were positively stained for ACE2, TMPRSS2, and furin (Figure 4). The ductal epithelium had positive signals for ACE2 (Figure 4A arrow) and TMPRSS2 (Figure 4B arrow), but not furin (Figure 4C arrow). The saliva of the ductal cavity had positive staining for ACE2, TMPRSS2, and furin. N-EL staining was completely negative in the submandibular glands.

### 2.2. Database Analysis

A total of 59 probes, including the serpin keyword, were found in the datasets. The database contains the TMPRSS2 inhibitor plasminogen activator inhibitor type 1, the furin inhibitor serpin B8, and the N-EL inhibitor leukocyte elastase inhibitor.

### 2.3. TMPRSS2, Furin, and N-EL Protein Expression

**Figure 5:**
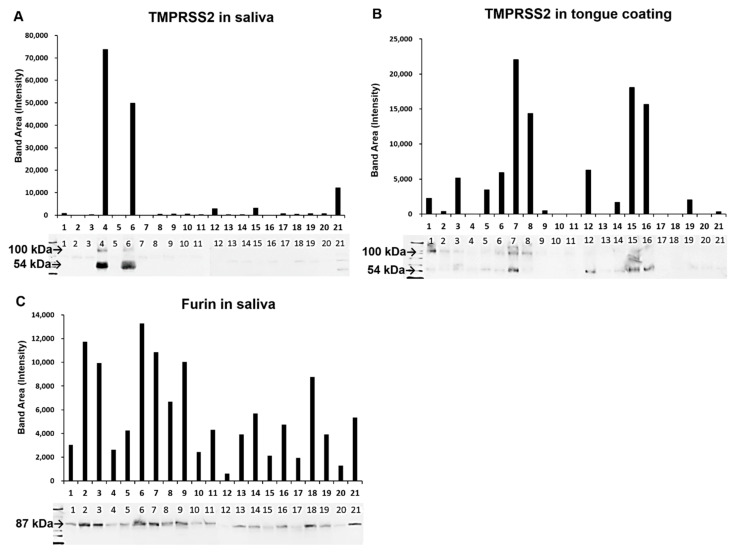
Band signals showing the protein expression levels of TMPRSS2 (**A**,**B**) and furin (**C**) via Western blot analysis, and a graph showing the intensity of the band area determined using Image J. TMPRSS2, transmembrane protease serine 2.

Western blotting (WB) was conducted on protein extracts from the saliva and tongue coating. A 54 kDa signal matching the molecular weight of full-length TMPRSS2 was detected (Figure 5A,B). No 26 kDa signals were observed for the cleaved serine protease domain. However, ~100 kDa signals were also noted. TMPRSS2 signals were not detected in four saliva and seven tongue coating samples, but no case for TMPRSS2 was negative in both the saliva and tongue coating. In the saliva and tongue coating samples, the median TMPRSS2 expression values were 371.678 and 2260.033 for men, and 584.506 and 977.578 for women, respectively. No significant gender-related difference was noted between TMPRSS2 in the saliva and tongue coating. There was no correlation between saliva and tongue coating TMPRSS2 expression. In the saliva, the 87 kDa signal for furin was observed in all cases (Figure 5C), but no furin was detected in tongue coating. The mean ± SE for saliva furin was 7203 ± 1222 for men and 3833 ± 774 for women, and the difference between the gender groups was significant (*p* = 0.033). N-EL protein was not detected in the saliva or tongue coating.

### 2.4. ACE2, TMPRSS2, and Furin Expression in Cultured Taste Buds

**Figure 6:**
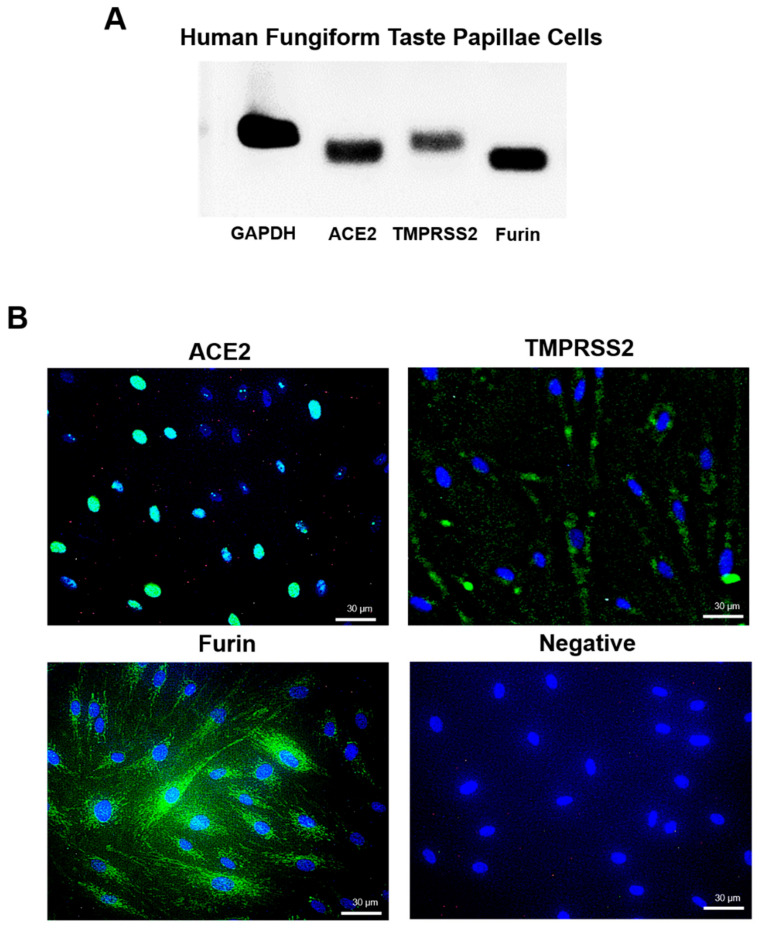
Expression and localization of ACE2, TMPRSS2, and furin mRNA and protein in human fungiform papillae taste cells. (**A**) RT-PCR analysis showing glyceraldehyde 3-phosphate dehydrogenase (GAPDH), ACE2, TMPRSS2, and furin in human fungiform papillae taste cells. (**B**) Immunochemical staining for ACE2 (green), TMPRSS2 (green), furin (green), and the nucleus (blue) in human fungiform papillae taste cells. The negative control section was stained with non-immunized rabbit IgG. Scale bar = 30 µm. ACE2, angiotensin-converting enzyme 2; TMPRSS2, transmembrane protease serine 2.

Amplified products corresponding to glyceraldehyde 3-phosphate dehydrogenase (GAPDH), ACE2, TMPRSS2, and furin transcripts were detected in RT-PCR analysis of samples derived from human fungiform papillae taste cells (Figure 6A). Immunocytochemical staining demonstrated that ACE2 was localized in the nucleus of human fungiform papillae taste cells. TMPRSS2 was weakly expressed and localized in the cytoplasm. Furin was localized in the cytoplasm and expressed intensely. The immunocytochemistry results agreed with the mRNA expression level results (Figure 6B). No staining was detected in the nucleus or cytoplasm of the negative control; only nuclear 4′, 6-diamidino-2-phenylindole (DAPI) staining was observed.

## 3. Discussion

More extensive ACE2 expression was observed in the epithelial surface and horny layer of the tongue than in the gingiva. In the oral cavity, ACE2 is reported to be predominantly localized to the basal cells of the non-keratinized stratified squamous epithelium [4]. In this study, ACE2 expression was stronger in the basal layer than in the epithelial surface and horny layers, which is in agreement with previous reports. However, ACE2 expression in the upper layer of the stratified squamous epithelium was diffuse and varied among individuals. ACE2 expression is affected by female hormones [15], salt [16], and smoking [17], among other factors. Since the tongue is an organ in the oral cavity that is exposed to these factors, they may affect ACE2 expression in the tongue. In detached upper surface layer cells, ACE2 was localized in the cell membrane, and no positive signals were observed in the nucleus. The extracellular domain of ACE2 has a binding site for SARS-CoV-2 [18]. Therefore, the infection potential of the tongue may be limited to superficial shedding cells. Wang et al. reports proliferation of SARS-CoV in exfoliated epithelial cells in saliva [19]. This report indicates that infected cells are contained in the oral cavity, suggesting that aspiration of these cells may be involved in lower respiratory tract (LRT) infection [19].

TMPRSS2 was consistently expressed in the cell membrane of the dorsal tongue squamous epithelium. TMPRSS2 has been reported to be expressed in the squamous epithelium of the tonsils [20] as well as other glandular tissues [21]. However, this is the first report of TMPRSS2 expression in the keratinized stratified squamous epithelium of the dorsal tongue. Conversely, furin was mainly expressed in the basal layer of stratified squamous epithelium. Previous studies [22] using healthy mucosa from the margins of squamous cell carcinoma report similar results in terms of expression intensity. Furin localization was observed in the deeper parts of the stratified squamous epithelium, but not the superficial layer. Therefore, it can be inferred that TMPRSS2 has a greater role in infection. ACE2–TMPRSS2 coexpression is a potential target for SARS-CoV-2 infection [23], and the squamous epithelial cells of the dorsal tongue are theoretically at risk of SARS-CoV-2 infection. However, in the dorsal tongue, the squamous epithelium is thick due to stratification of the cells. Therefore, it is unclear as to whether the cells in the superficial layer can infect subepithelial cells. Although the distribution and amounts of ACE2 in organs are strongly associated with the clinical manifestations of COVID-19 [12], there are few reports of COVID-19 patients showing symptoms such as glossodynia [24]. However, shed cells from the stratified squamous epithelium tended to have stronger ACE2 and TMPRSS2 expression. Furthermore, these exfoliated cells are likely to be mixed with bacteria on the surface of the tongue. Tu et al. detect SARS-CoV-2 in the dorsum of the tongue of COVID-19 patients with a detection sensitivity of 89.8% [25]. The tongue coating contains SARS-CoV-2 and removal of the tongue coating reduces the risk of the dentist’s exposure to the virus and formation of aerosols.

Taste buds were found in the fungiform papillae of stratified tongue squamous epithelium, and taste cells were positive for ACE2, TMPRSS2, and furin via immunohistochemistry. In addition, presence of ACE2, TMPRSS2, and furin mRNAs was confirmed in cultured taste bud cells. There are currently no reports discussing ACE2 expression in taste cells. However, angiotensin-converting enzyme 1 (ACE1) is observed in mouse taste cells, and it has been reported that taste function may be regulated by both locally produced and circulating angiotensin II (AngII) [26]. The presence of the renin–angiotensin system in taste cells has been suggested, and ACE2 expression may therefore be involved in taste. Furthermore, COVID-19 involves an olfactory abnormality, and it has been reported that ACE2 and TMPRSS2 expression in olfactory epithelial supporting cells and stem cells may be involved in the mechanism underlying the olfactory abnormality in COVID-19 [27]. Loss of olfaction is one of the causes of dysgeusia. The cause of COVID-19 dysgeusia may be related to the decreased expression of ACE2 resulting from SARS-CoV-2 infection. Moreover, many nerve fibers are found in the taste buds. Further careful research is needed to clarify the mechanism of dysgeusia induced by SARS-CoV-2 infection.

TMPRSS2 and furin, but not N-EL, were detected in saliva. TMPRSS2 and furin were detected in the mucus of the salivary duct by immunohistochemistry, but not N-EL. This is the first report to suggest that the TMPRSS2 and furin present in saliva are produced by salivary glands. A gender-based difference was observed in the presence of furin, and men tended to have higher expression than women did. Plasma furin levels are higher in men than in women, indicating that furin expression may be higher in men [28]. Moreover, furin was detected in the saliva in all cases, but TMPRSS2 varied from being undetectable to extremely high concentrations, and large individual differences were also observed. In addition, the serine protease region of TMPRSS2 is identified as a band of 54 kDa [29], suggesting that salivary TMPRSS2 exhibits protease activity, and as a band of ~100 kDa, suggesting possible glycosylation (Figure 5). TMPRSS2 is androgen-regulated, and androgen receptors are present on the ductal epithelium and serous cells of the salivary gland, which may affect TMPRSS2 production in saliva. Since COVID-19 has been reported to have a higher mortality rate in men than women [30], it may be worth exploring how these proteases differ between the sexes.

We reported, for the first time, the presence of full-length TMPRSS2 protein containing serine protease in the tongue coating. Furthermore, some cases were negative for salivary TMPRSS2 while all were positive for tongue coating TMPRSS2. This indicates that TMPRSS2 protein may be expressed in the tongue coating and saliva. In addition, furin was not detected in tongue coating but was detected in saliva. Interestingly, there were no furin-positive cells on the surface of the dorsal tongue squamous epithelium. Conversely, TMPRSS2 was strongly expressed in the upper layer of the stratified squamous epithelium. These findings suggest that the tongue coating protease is derived from the stratified squamous epithelium of the dorsal tongue and saliva. Immunostaining showed TMPRSS2 expression in stratified squamous epithelium in all cases, but some cases were negative for tongue coating TMPRSS2. This suggests that TMPRSS2 exhibits activity at a level that cannot be detected by WB [31], and thus a method for detecting trace amounts is required. It is presumed that all cases will be TMPRSS2-positive when examined by enzyme-linked immunosorbent assay.

Protease inhibitors were extracted by database analysis, and 59 molecules were selected as candidate protease inhibitors. These included plasminogen activator inhibitor type 1, which may inhibit TMPRSS2 activity [32], and serpin B8, which has been reported to be a furin inhibitor [33]. A balance between proteases and protease inhibitors is reported to be another requirement for viral infection [34]. In addition, RNase in the saliva acts as a resistance factor against RNA viruses. Saliva also contains components such as lactoferrin and IgA, which have antibacterial and antiviral effects [35,36]. Therefore, although there are factors for SARS-CoV-2 infection in the oral cavity, the actual situation in the oral cavity is extremely complicated.

In gingival epithelial cells, not only were ACE2, TMPRSS2, and furin expressed in the sulcular epithelium and periodontal pocket epithelium, but also N-EL in the neutrophils infiltrating the epithelium. N-EL aggravates pneumonia and promotes the spread of infection in SARS [36], but thus far, there have been no similar reports for SARS-CoV-2. However, N-EL may be a risk factor for the spread of infection [37].

Human papilloma virus (HPV) has been previously detected in the periodontal pocket epithelium by in situ hybridization [38]. Therefore, the possibility of viral entry into the periodontal pocket epithelium cannot be ruled out. The periodontal pocket epithelium is formed by the extension of the sulcular epithelium. Therefore, there may be a risk of SARS-CoV-2 infection via the periodontal pocket epithelium. It is necessary to investigate COVID-19-related molecules targeting periodontal pocket epithelium.

## 4. Materials and Methods

### 4.1. Pathological Sample Selection

Formalin-fixed paraffin-embedded blocks of pathological samples were obtained from Kanagawa Dental University Hospital, which were dissected in 2018–2020. Since the 15 tongue samples were mainly diagnosed with hemangioma of the dorsal tongue, no dysplastic changes, inflammation, or fungal infection was detected. The patients were 10–80 years of age, with a mean of 53.6 years. Six of the samples were from men, and nine from women. The 16 gingival samples included the sulcular and gingival epithelium, and these samples had mild to severe inflammation due to epulis. The patients were 20–91 years of age, with a mean of 51.88 years. Six of the samples were from men, and ten from women. All tissue samples were used according to protocols approved by Kanagawa Dental University IRB (No. 650, approved on 01 April, 2020).

### 4.2. Saliva Collection and Tongue Coating Sampling

Salivary samples were collected from patients and staff of the Kanagawa Dental University Yokohama Clinic from 06 April, 2020 to 08 April, 2020 after obtaining informed consent. We selected subjects who had no symptoms of COVID-19 for more than 2 weeks. Samples were collected from 9 a.m. to noon. Unstimulated saliva was collected by passive drooling into a 50 mL polypropylene tube for 5 min. The saliva collected was immediately cooled on ice. Next, the collected samples were spun in a hybrid high-speed refrigerated centrifuge (6200; Kubota, Tokyo, Japan) at 3000× *g* for 5 min at 4 °C. After centrifugation, the supernatant was frozen at −80 °C until analysis. After removing saliva from the dorsal tongue of the patient with air, any tongue accretion was carefully removed with five scratching strokes (approximately 1 cm long) using a tongue brush (Oral Care Inc., Tokyo, Japan) from the posterior-center area of the dorsal tongue. The tongue brush with tongue coating was placed in a 50 mL polypropylene tube and stored at −80 °C until analysis. The patients were 24–69 years of age, with a mean of 45.6 years. Eleven of the samples were from men, and 10 from women. All tissue samples were used in accordance with Kanagawa Dental University IRB-approved protocols (No. 651, approved on 01 April, 2020).

### 4.3. Immunohistochemistry

Immunohistochemical (IHC) staining was conducted via the enzyme antibody method. Primary antibodies and antigen retrieval information are shown in Table 2. After the paraffin removal and dehydration of sections, antigen retrieval was performed by microwaving for 15 min. Sections were treated with primary antibodies and incubated at 37 °C for 1 h. They were then incubated with anti-mouse and anti-rabbit secondary antibody-labeled peroxidase (Nichirei, Simple-Stain MAX-PO Multi, Tokyo, Japan) at 22 °C for 30 min. The color was developed with 0.05% diaminobenzidine and hydrogen peroxide, and the tissue was counterstained with Mayer’s hematoxylin.

Submandibular gland samples were used as a positive control, and lymph node samples as a negative control. For these controls, samples from five patients with squamous cell carcinoma were used.

The specificity of the primary antibodies was demonstrated in the abolished test. Recombinant proteins (10 μg/mL) and primary antibodies for ACE2, TMPPRS2, and furin were added to the sections. Lymph node B cells were used as a negative control and PBS was used as a negative control for the secondary antibody. Section staining was scored based on a scale of 0–2 (0, no staining; 1, definite staining of some cells, i.e., <30% of cells; and 2, definite staining of a majority of cells, i.e., >70% of cells). We used the counting method reported by Lucas [38], with some modifications. The epithelium surface, horny layer, and spinous–basal cell layer were evaluated individually. A positive ACE2 reaction signal was classified as nuclear-cytoplasmic or membrane-cytoplasmic. The TMPRSS2 localization pattern was also evaluated as either membrane only or mixed membrane-cytoplasmic.

### 4.4. Western Blot Analysis

Tongue plaque coating was homogenized with RIPA Buffer (Nacalai Tesque, Kyoto, Japan) and centrifuged at 4 °C and 1700× *g* for 10 min. After centrifugation, the supernatant was collected. The saliva was used as an undiluted solution and was centrifuged at 300× *g* for 15 min at 4 °C. Each protein concentration was determined using a NanoDrop™ One spectrophotometer (Thermo Fisher Scientific, Waltham, MA, USA). Supernatant (65 µL) was mixed with NuPAGE LDS Sample Buffer (Novex, Darmstadt, Germany) and NuPAGE Sample Reducing Agent (Novex) and incubated at 96 °C for 5 min.

Each sample was adjusted to the same protein concentration and was applied to a 15% polyacrylamide gel (Wako Pure Chemical Industries, Osaka, Japan), separated by sodium dodecyl sulfate-polyacrylamide gel electrophoresis (SDS-PAGE), and transferred to a polyvinylidene fluoride membrane (Merck, Branchburg, NJ, USA). The antibodies used in the aforementioned immunohistochemical analysis were also used in the WB analysis. Secondary antibodies (Dako, Glostrup, Denmark) were diluted 1:2000 in TBST containing 5% non-fat dry milk. Immunoreactive signals were detected with a secondary antibody labeled with alkaline phosphatase and visualized using Luminate Forte Western HRP Substrate (Millipore, Billerica, MA, USA). WB images were acquired with a CCD camera (LAS 3000, Fujifilm, Tokyo, Japan). Each marker was exposed simultaneously under the same conditions (21 samples). The intensity of bands in the Western blotting was calculated using Image J analysis software (NIH, Bethesda, MD, USA).

### 4.5. Human Fungiform Papillae Taste Cell Culture

Human fungiform papillae taste cells (T4105-c, Applied Biological Materials, Richmond, Canada) were cultured in dishes pretreated with Applied Cell Extracellular Matrix in Prigrow X Medium, containing 10% fetal bovine serum (both from Applied Biological Materials) and penicillin/streptomycin, at 37 °C under 5% CO_2_, according to the manufacturer’s instructions. The culture medium was replaced every 3 days.

### 4.6. Gene Expression Analysis and Immunofluorescence Staining of Papillae Taste Cells

Total RNA was extracted from cultures using TRIzol reagent (Life Technologies, Carlsbad, CA, USA) and purified using Direct-zol™ RNA MiniPrep Kits (Zymo Research Co., Irvine, CA, USA) when the cells had reached 80% confluence, according to the manufacturer’s protocol. The concentration of purity of the RNA was determined using a NanoDrop™ One spectrophotometer (Thermo Fisher Scientific, Waltham, MA, USA). Purified RNA was stored at −80 °C. Purified RNA was treated with DNase I reagent (Invitrogen, Carlsbad, CA, USA) and converted into complementary DNA (cDNA) using the SuperScript^®^ VILO™ cDNA System Kit (Invitrogen), as outlined by the manufacturer. Total RNA (1 µg) was used, and reverse transcription was performed with a final reaction volume of 20 µL, according to the manufacturer’s instructions. cDNA samples were stored at −20 °C until use. RT-PCR was conducted with the StepOne Plus Real-Time PCR System (Applied Biosystems, Waltham, MA, USA) using the FastStart DNA Master SYBR Green I Kit (Roche Applied Science, Mannheim, Germany), according to the manufacturer’s instructions. Gene expression of the following genes was visualized: ACE2 (forward: ACAGTCCACACTTGCCCAAAT and reverse: TGAGAGCACTGAAGACCCATT), TMPRSS2 (forward: CAAGTGCTCCAACTCTGGGAT and reverse: AACACACCGATTCTCGTCCTC), and furin (forward: TCGGGGACTATTACCACTTCTG and reverse: CCAGCCACTGTACTTGAGGC). GAPDH (forward: GGAGCGAGATCCCTCCAAAAT and reverse: GGCTGTTGTCATACTTCTCATGG) was used as the housekeeping gene. The primers were validated by performing a melting temperature analysis and inspecting the DNA bands via 1.5% Tris-borate buffer agarose gel electrophoresis using ethidium bromide staining.

The taste bud cells were fixed with 4% paraformaldehyde for 5 min and air-dried. The cells were blocked in 10% rabbit serum for 30 min at 22 °C and then incubated with primary antibodies for 1 h at 22 °C. After three washes with PBS (5 min each), the cells were incubated with Alexa Fluor 488-labeled polyclonal goat anti-rabbit IgG (Abcam, Cambridge, UK) in PBS at a dilution of 1:500 for 1 h at 22 °C followed by 4′,6-diamidino-2-phenylindole (DAPI) (Vector, CA, USA) for nuclei staining. For the negative control experiments, PBS was used instead of the primary antibody. Immunofluorescent signals were detected using Biozero BZ-8000 (Keyence Co., Osaka, Japan).

### 4.7. Database Analysis

To explore potential serine protease inhibitors in human saliva, we analyzed publicly available data. The dataset GSE14245 at Gene Expression Omnibus repository included the gene expression data for human saliva.

### 4.8. Statistical Analysis

TMPRSS2 expression in saliva and tongue coating was compared using the Mann–Whitney U test, as a Kolmogorov–Smirnov test showed that the data were not normally distributed. As the salivary furin data were normally distributed, furin expression was compared using an unpaired t-test. The relationship between the expression of TMPRSS2 and furin was analyzed using Spearman rank correlation coefficient. The significance level was set at 5%. All analyses were performed with the statistical software package IBM SPSS Statistics Version 24 (IBM Co., Armonk, NY, USA).

## 5. Conclusions

ACE2 and TMPRSS2 are the determining factors for SARS-CoV-2 infection that were identified in the oral cavity. In particular, it was considered that the expression of these molecules in the taste buds would allow elucidation of the mechanism by which COVID-19 symptoms such as taste disorders manifest. Furthermore, taste buds are theoretically thought to be the cells that influence SARS-CoV-2 invasion.

Tongue coating contains cells that coexpress ACE2 and TMPRSS2 and abundantly expressed TMPRSS2, which could theoretically be an infection-promoting factor. The origin of the tongue coating protease was suggested to be deciduous cells of the stratified squamous epithelium and saliva. These findings suggest that the tongue coating may be less susceptible to the effects of salivary protease inhibitors. Therefore, it can be inferred that removing the tongue coating would reduce the risk of dental workers being exposed to the virus, and the protease inhibitor in saliva may easily reach the tongue surface. Further research is required to support this suggestion.

The periodontal pocket epithelium is formed by the extension of the sulcular epithelium. The sulcular epithelium coexpresses ACE2 and TMPRSS2, and since the cell layer is thin, we believe that there is a high risk of internal infection. Therefore, it is suggested that the periodontal pocket epithelium may be a focal point of infection.

## Figures and Tables

**Table 1:** Expression level of ACE2, TMPRSS2, and furin in the tongue and gingiva.

	Gingiva (n = 16)			Tongue (n = 15)		
**Expression Levels**	0	1	2	0	1	2
**ACE2 Expression**						
**Surface Layer**	100.0% (16/16)	0.0% (0/16)	0.0% (0/16)	46.7% (7/15)	40.0% (6/15)	13.3% (2/15)
**Horny Layer**	68.8% (11/16)	25.0% (4/16)	6.3% (1/16)	13.3% (2/15)	73.3% (11/15)	13.3% (2/15)
**Spinous–Basal Cell Layer**	0.0% (0/16)	0.0% (0/16)	100.0% (0/16)	0.0% (0/15)	60.0% (9/15)	40.0% (6/15)
**TMPRSS Expression**						
**Surface Layer**	0.0% (0/16)	0.0% (0/16)	100.0% (16/16)	0.0% (0/15)	0.0% (0/15)	100.0% (15/15)
**Horny Layer**	6.3% (1/16)	50.0% (8/16)	43.8% (7/16)	0.0% (0/15)	40.0% (6/15)	60.0% (9/15)
**Spinous–Basal Cell Layer**	0.0% (0/16)	0.0% (0/16)	100.0% (16/16)	0.0% (0/15)	0.0% (0/15)	100.0% (15/15)
**Furin Expression**						
**Surface Layer**	100.0% (16/16)	0.0% (0/16)	0.0% (0/16)	100.0% (15/15)	0.0% (0/15)	0.0% (0/15)
**Horny Layer**	87.5% (14/16)	12.5% (2/16)	0.0% (0/16)	100.0% (15/15)	0.0% (0/15)	0.0% (0/15)
**Spinous–Basal Cell Layer**	0.0% (0/16)	31.3% (5/16)	68.8% (11/16)	0.0% (0/15)	66.7% (10/15)	33.3% (5/15)

(·), indicates the number of cases; 0, no staining; 1, definite staining of some cells, i.e., <30% of cells; 2, definite staining of a majority of cells, i.e., <70% of cells.

**Table 2:** Antibodies used for immunostaining and Western blotting.

Antibody	Clone	Supplier	Dilution for IHC	Dilution for WB	Retrieval
ACE2	HPA000288	Abcam	200	1000	Citrate buffer (pH 6)
TMPRSS2	EPR3861	Abcam	1000	1000	Tris-EDTA (pH 9)
Furin	EPR14674	Abcam	500	1000	Citrate buffer (pH 6)
Elastase	ab68672	Abcam	500	5000	Tris-EDTA (pH 9)

## Supplementary Materials

Supplementary materials can be found at https://www.mdpi.com/1422-0067/21/17/6000/s1.

## Abbreviations

ACE2	Angiotensin-converting enzyme 2
AngII	Angiotensin II
DAPI	4′,6-Diamidino-2-phenylindole
HPV	Human papilloma virus
LPT	Lower respiratory tract
N-EL	Neutrophil-elastase
SP	Spike protein
TMPRSS2	Transmembrane protease serine 2
WB	Western blotting
IHC	Immunohistochemical
