# Supplementary material for: Existence of SARS-CoV-2 Entry Molecules in the Oral Cavity
Source: Int J Mol Sci. 2020 Aug 20;21(17):6000. doi: 10.3390/ijms21176000 (PMC7503451; doi:10.3390/ijms21176000)
Supplement: Supplementary file 1 [file ijms-21-06000-s001.zip › 20.08.12_ijms_COVID-19_Suppl_Mate.pdf]

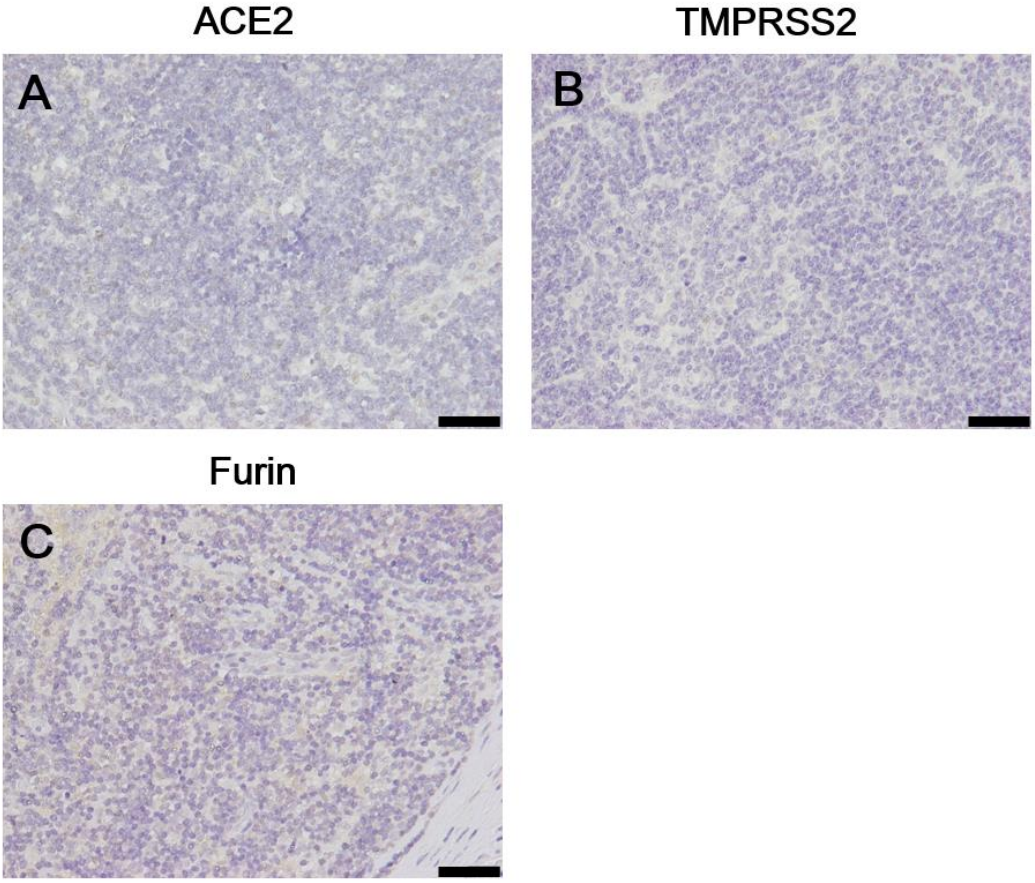

Figure S1. ACE2, TMPRSS2, and furin immunohistochemistry for lymph node B cells as negative control. In negative control sections comprising lymph node B cells, expression of ACE2 (A), TMPRSS2 (B), and furin (C) is completely absent. Scale bar = 40 μm. ACE2, angiotensin-converting enzyme 2; TMPRSS2, transmembrane protease serine 2.

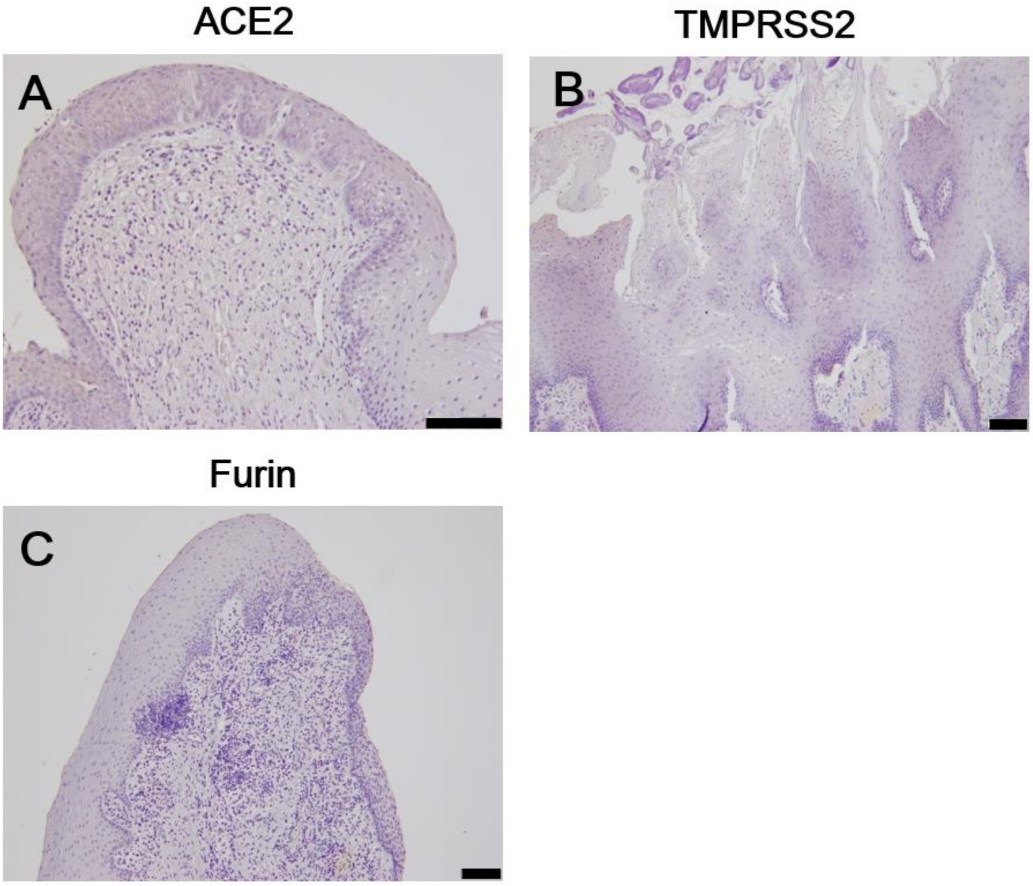

Figure S2. ACE2, TMPRSS2, and furin immunohistochemistry for representative negative controls, respectively. In negative control sections comprising fungiform papilla of the tongue epithelium, expression of ACE2 is completely absent (A). In negative control sections comprising tongue epithelium, expression of TMPRSS2 is completely absent (B). In negative control sections comprising gingival tissue, expression of furin is completely absent (C). Scale bar = 100 μm. ACE2, angiotensin-converting enzyme 2; TMPRSS2, transmembrane protease serine 2.
